# Adaptation in the visual cortex: a case for probing neuronal populations with natural stimuli

**DOI:** 10.12688/f1000research.11154.1

**Published:** 2017-07-27

**Authors:** Michoel Snow, Ruben Coen-Cagli, Odelia Schwartz

**Affiliations:** 1 Department of Neuroscience, Albert Einstein College of Medicine, Bronx, NY, 10461, USA; 2 Department of Systems and Computational Biology, Albert Einstein College of Medicine, Bronx, NY, 10461, USA; 3 Department of Computer Science, University of Miami, Coral Gables, FL, 33146, USA

**Keywords:** visual cortical neurons, visual adaptation, natural scenes, population coding

## Abstract

The perception of, and neural responses to, sensory stimuli in the present are influenced by what has been observed in the past—a phenomenon known as adaptation. We focus on adaptation in visual cortical neurons as a paradigmatic example. We review recent work that represents two shifts in the way we study adaptation, namely (i) going beyond single neurons to study adaptation in populations of neurons and (ii) going beyond simple stimuli to study adaptation to natural stimuli. We suggest that efforts in these two directions, through a closer integration of experimental and modeling approaches, will enable a more complete understanding of cortical processing in natural environments.

## Introduction

Visual adaptation, hereon simply referred to as adaptation, is the influence of past visual stimuli on the responses of neurons and on perception of the present. Though adaptation was first identified millennia ago ^1^, its principles and functional role are still not well understood.

The earliest experimental studies of neuronal-level adaptation effects used simple, artificial stimuli repeated over time, such as oriented bars and moving dots, a technique still commonly used. Simple repetitive stimuli such as these can result in striking perceptual after-effects. For instance, in the tilt after-effect, prolonged exposure to a particular orientation of a grating results in systematic biases in perception of the orientation of another grating presented later in time ^2^. Adaptation to stimulus features such as orientation and contrast can notably change the gain and tuning properties of neurons in early cortex as well as other response properties. These phenomena have been reviewed previously (see e.g. 3– 8). Here, we also start from this class of adaptation phenomena because they provide the springboard for the more recent developments that are the focus of this review. We then identify ways in which these effects are being reinterpreted and extended within the context of natural scenes and neuronal populations.

While we have gained considerable knowledge with the classical approach, it has some important limitations. First, adaptation effects are not limited to low-level image features such as orientation and contrast, as they have also been observed for higher-level visual content such as facial expression and for complex stimuli such as natural scenes ^9, 10^, with some studies focusing on influences across the cortical hierarchy (see e.g. 11). Furthermore, as compared to artificial stimuli, the visual inputs we receive from the natural environment have more complex temporal dynamics; for instance, we may view dynamically changing images in a given location ^12^ or entirely new scene structure owing to eye movements. Second, most experiments have probed adaptation in single neurons ^8^. Visual processing, however, relies on populations of neurons, and circuit-level effects of adaptation are poorly understood (but see recent progress ^13– 18^). As a consequence, we have a limited understanding of what aspects of neural adaptation are responsible for the observed perceptual effects, as we illustrate below. Third, computationally, despite recent advances ^19– 25^, we still lack a comprehensive model that can predict when adaptation will be recruited for arbitrary, natural stimuli or the degree to which it will occur. More generally, the link between the observable effects and the functional goals of adaptation has been elusive.

One promising approach to address these issues is based on the assumption that neural systems are sensitive to the statistical structure of stimuli in the natural environment over space and time and that adaptation reflects these statistics ^6, 7, 26– 32^. According to this view, to more fully understand and accurately model adaptation would require studying neural systems exposed to the natural environment. Here, we review recent literature that is taking studies of adaptation in exciting new directions, focusing on new experiments, analyses, and models that (i) are going beyond the single-neuron level and tapping into the neuronal population level and (ii) are using naturalistic scenes in lieu of simple, artificial stimuli.

For recent reviews covering other aspects of visual adaptation, such as timescales of adaptation, effects outside of the classical receptive field, inheritance across multiple stages of neural processing, potential neural mechanisms, and compensation for biological variation, see 8, 33. Adaptation may also be related to forms of plasticity that take place on different timescales such as perceptual learning and developmental processes, and studies of adaptation have extended beyond the traditional sensory domains to systems such as memory and action ^34– 39^. Although beyond the scope of this review, we believe that the framework that is emerging from the more limited set of studies we discuss here on natural scenes and population coding could, in the future, provide a conceptual bridge to those aforementioned domains.

## Adaptation in neuronal populations

Most neurophysiology studies of adaptation have focused on single neurons—more specifically, examining the average neural response across repeated trials of the same stimulus condition ^8^. However, some adaptation effects are revealed only by analyzing the activity of populations of neurons. For example, Benucci *et al.* ^40^ demonstrated a form of homeostasis across the neuronal population while recording simultaneously from tens of neurons. They adapted the population to a biased stimulus ensemble in which some orientations were presented more frequently than others. They found that after adaptation, despite the stimulus bias, neurons with different orientation preferences, on average, had the same response level across the ensemble. Such population data offer a richer test bed for models of adaptation ^19, 20^ and have the potential to link to perceptual phenomena.

Here we focus, in particular, on adaptation as it affects the variability of neural activity. It is well known that neural responses fluctuate substantially across trials ^41, 42^ and that such fluctuations are shared between neurons, as quantified by “noise correlations” ^43^. This variability can strongly influence information in neuronal populations and, ultimately, perception ^44– 51^. The structure and stimulus dependence of cortical variability have been thoroughly characterized ^52– 58^, and recent studies have begun to examine how it is affected by adaptation.

Benucci *et al*. ^40^ reported that, in their experimental paradigm, adaptation did not affect the overall level of noise correlations. The adapted neurons exposed to the biased stimulus ensemble maintained the same degree of average correlation as the unadapted neurons did to the uniform stimulus ensemble, regardless of the similarity between the preferred orientation of the neurons and the biased orientation. This result was at odds with a previous study ^59^ based on the traditional adaptation paradigm of prolonged exposure to a single stimulus (a grating with fixed orientation, as opposed to a biased ensemble). Gutnisky *et al.* ^59^ reported an overall reduction of noise correlations in primary visual cortex (V1) after adaptation. The strength of the reduction depended on the relative orientation preference of the neurons and the orientation of the adapter. They also speculated that these effects could increase population information about stimulus orientation.

A more recent study has also reported evidence for decorrelation ^60^, primarily for pairs of neurons in marmoset middle temporal visual area (MT), whose preferred direction of visual motion was similar to the adaptor. The authors took a more direct approach to assessing the consequences of adaptation for population information. They used a decoding-based analysis to read out, on a trial-by-trial basis, the direction encoded by the population. They found no impact of adaptation on decoder performance, i.e. no change in the percentage of cases where the decoder’s output matched the true test direction. However, the distribution of errors was asymmetrical, and the decoder was on average biased in a manner consistent with the perceptual direction after-effect ^61, 62^, i.e. the decoded direction was repelled away from the true direction by up to 5 degrees when the adapter was around 60 degrees away from the test. Zavitz *et al.* ^60^ also found that noise correlations had a minimal effect on the decoder’s performance; the direction after-effect was attributed mainly to the effects of adaptation on response gain, consistent with previous models in the temporal ^7^ and spatial ^63, 64^ domains. Another recent study ^65^ has focused on population-level adaptation in rat barrel cortex, reporting that adaptation generally increases noise correlations but increases single-neuron information even more. The net effect is that adaptation increases information at the population level around the adapter.

The heterogeneity of effects reported in these studies is indicative of a number of caveats that should be considered when studying population coding. For instance, the conclusion of Gutnisky *et al.* ^59^, that adaptation increases information, was based on simulating populations with artificially constructed tuning curves and covariances, a method that is prone to mis-estimation of information ^49, 51^. Benucci *et al.* ^40^ analyzed noise correlations only at a coarse level, and it is possible that a decoding-based approach could reveal a role of noise correlations despite their relatively small change, on average, across the population. The decoder-based analysis of Zavitz *et al.* ^60^ and Adibi *et al.* ^65^ is a safer route to address the effects of adaptation on stimulus discrimination performance; however, it has recently become clear that correlations may have a substantially different impact on information for small populations of few tens of neurons versus larger populations with size comparable to the number of neurons presumably involved in solving the perceptual task ^51^. It is also important to keep in mind that decoding is always task dependent and conclusions drawn from decoding-based analyses are specific to the task considered (e.g. discrimination of visual orientation in V1 or motion direction in MT). Adaptation-induced changes in the population code may have different functional relevance when considering different tasks or computational goals, a point we explore further below.

The field clearly needs a much more extensive and systematic study of population-level adaptation and its relation to perception. We suggest that this effort could benefit from leveraging recent developments in the broader field of neuronal population coding: 1) simultaneous recording from larger populations of hundreds (which is feasible with current technology ^53^) or thousands of neurons (which might soon become feasible ^66^), 2) decoding-based analysis of population data, which is becoming commonplace in other studies of visual processing besides adaptation ^49, 67– 70^ (please see 47, 71 for broader reviews on population decoding), and 3) theories of population coding in the context of well-specified computational goals ^72, 73^. While this section has focused on the first two elements above, in the next section we discuss recent proposals for the computational goals of adaptation that go beyond discrimination tasks with simple stimuli.

## Adaptation to natural scenes

While we have learned a lot from studying adaptation to simple stimuli, and these continue to be useful as benchmarks for systematic manipulation, replication, and comparison, there has been growing interest in understanding adaptation for more naturalistic stimuli. This interest is because of the difficulty in extrapolating from simple stimuli how the brain adapts to stimuli it encounters in the natural environment. There is also reason to expect that aspects of neural responses are tuned to the properties of the visual environment, as has been demonstrated in many areas of visual processing ^26, 29, 32, 74, 75^. Some studies have pushed forward the hypothesis that neural properties are constrained by task-related goals: for example, Burge and Geisler ^76^ used natural scenes to develop an ideal observer for speed estimation and showed that this closely matched human performance. Other studies have focused on deriving models of visual neurons with task-independent goals, such as efficient coding. In this section, we first discuss the use of natural stimuli in experimental paradigms of adaptation. We then discuss computational models of adaptation motivated by the structure of visual scenes. Finally, we note new techniques which potentially can be applied to adaptation in the near future.

Many studies of adaptation have focused on synthetic stimuli owing to the inherent complexity of natural stimuli and the difficulty of parsing specific aspects of these stimuli to create controlled experiments ^28^. Recently, there has been a trend of experimental design using more naturalistic stimuli, with the main focus on using static natural images ^77– 82^. The use of natural stimuli can often provide similar benchmarks to synthetic stimuli. For example, the perceptual tilt after-effect seen with synthetic stimuli is still observed with natural images (chosen according to their dominant orientation), but to a lesser degree ^77, 78^. Repulsive adaptation effects have also been studied for faces and have been reported for higher-level properties such as openness of the scene ^9, 83^. Taking a different approach, in an elegant reversal of the conventional design, instead of starting from a gray screen and then testing the effect of adapting to an oriented contrast grating, Haak *et al.* ^84^ used an alternate reality system to remove an orientation from subjects’ otherwise natural visual input, continuously, over multiple days. The overall strength of the adaptive effects peaked after the first day, then declined, but increased again more slowly as the days progressed. Each of their testing paradigms had its own set of peaks and troughs, with the strength of effects changing within individual sessions and across progressive days. They concluded that the variations in adaptive effects were due to multiple neural mechanisms operating at different time scales, in line with their earlier work ^85^ and related to results seen for synthetic stimuli (for review, see 8).

Computational models have also started to incorporate more aspects of natural scenes. We first discuss theoretical approaches for modeling the functional role of adaptation. We then focus on how the form and parameters of such models may be constrained and learned from natural scenes. One early hypothesis was that adaptation implements efficient coding principles (see, for example, the discussion in 4, 7). Examples of this perspective range from a reduction of metabolic cost ^86^ to improved signal-to-noise ratio ^22^, enhanced information transmission ^87, 88^, redundancy reduction ^19, 23, 25, 26, 75, 89^, and complementary directions of probabilistic inference in generative models of the environment ^20, 24^. Foundational work has related changes in V1 population-level activity over long time scales (development) to learning a well-calibrated prior for natural stimuli ^36^. Along with other similar work, this has provided a normative framework of how response variability and noise correlations depend on features of the visual inputs ^90^, and recent findings indicate that a similar approach could provide a new view of population-level adaptation on relatively shorter timescales (minutes to hours ^91^).

Another hypothesis states that adaptation serves to enhance stimulus salience, which may be related to the notion of reducing redundant information. Salience has been studied extensively in the spatial context domain (e.g. 92– 94). In particular, computational modeling and experimental tests have supported the hypothesis that V1 forms a salience map corresponding to the breakdown of homogeneity of the input ^92, 93, 95, 96^. In the temporal domain, salience may be viewed as novelty detection, postulating that adaptation serves to enhance neural responsivity to stimuli that are unexpected, e.g. stimuli that differ significantly from the adapter. This relates to ideas of predictive coding ^21^ and to literature on the mismatch negativity in evoked potentials, which is more prominent in the auditory domain ^97– 100^. Recently, experimental groups have started to perceptually test the hypothesis that adaptation enhances saliency ^101– 103^.

In recent work, following some of our earlier approaches on spatial context modeling ^104^, we have proposed, in the temporal domain, that adaptation effects may be explained as probabilistic inference in a generative model of the statistical dependencies in natural movies ^20^. In this framework, adaptation also reduces statistical redundancies that are induced by the stimuli. The redundancy reduction is achieved by adjusting the strength of a divisive normalization signal based on inference in the model about whether stimuli in the present and past are deemed statistically dependent. This constitutes a generalization of earlier work on redundancy reduction in still images and divisive normalization ^25, 105^, and relates to previous work on salience as a breakdown of statistical homogeneity ^93^.

Divisive normalization refers to a non-linear computation, whereby the response of a given neural unit is divided by the activity of other neural units ^106^. It has been termed a canonical computation in cortex ^107^ and has been shown to be consistent with adaptation phenomena in a range of other cortical areas, such as the auditory cortex, olfactory system, visual attention, and integration of multisensory information ^4, 8^. A mechanism for divisive normalization in the context of adaptation has been proposed recently, in which adaptation adjusts the strength of the interactions between model neurons (specifically, the weights of a divisive normalization signal) to homeostatically maintain the products of responses of pairs of neurons ^19^. Both modeling frameworks ^19, 20^ replicated aspects of the main adaptation phenomena, namely suppression and repulsion at the single-neuron level as well as equalization of population responses, and they both could explain the tilt after-effect.

However, although learned with natural scenes, the adaptation model of Snow *et al*. ^20^ (as well as other models inspired by or learned with natural scenes) have typically been tested experimentally only with simple stimuli. While an important first step, simple stimuli lack the richness of natural stimuli, with which such models are learned. This constitutes a limitation not only for testing our models but also, more broadly, when a variety of cortical properties appear to be attuned to the inherent properties of natural scenes ^76, 108^. Testing computational models with natural stimuli offers a much richer test set. More importantly, one can study neural or perceptual responses to natural stimuli in the context of the computational models and their predictions.

Testing computational models derived from natural scenes with natural scene stimuli has been emerging in other domains. For instance, in studying spatial (rather than temporal) context effects, Coen-Cagli *et al*. ^109^ derived a model of V1 from the statistics of static natural scenes. Using probabilistic inference in a generative model of spatial dependencies in images, the model made the specific prediction that when visual inputs to the receptive field center and surround of a neuron were deemed statistically dependent according to the model, this resulted in more recruitment of surround suppression to reduce the dependency. Through close interplay between the modeling and neurophysiology experiments using natural scenes in V1, this study suggested that surround suppression in cortical neurons is gated by the statistical similarity of center and surround stimuli. These directions can be extended to adaptation models and to more natural stimuli for adaptation, such as movies that incorporate motion in the environment and eye movements. There is also the potential to extend such approaches to perceptual studies (e.g. in the spatial domain ^110^).

We have thus far focused largely on unsupervised learning in natural scenes and how such approaches may be used to build and test models of adaptation in early cortex. There are also other potential routes to modeling neural adaptation based on learning with natural scenes. Recently, there have been exciting advances in an area of machine learning known as deep convolutional neural networks. Deep convolutional neural networks consist of a hierarchy of layers that learn progressively more complex structure in images, inspired by the hierarchical organization in the visual cortex. Recent advances in the field have focused on supervised task-based learning approaches that discriminate between a large ensemble of labeled images. Modern versions of deep convolutional neural networks have led to state-of-the-art results in scene recognition in computer vision ^111, 112^. These approaches have in turn been recently applied within the neuroscience community, with the goal of capturing cortical processing in higher visual areas, beyond V1. There has been some indication of success relative to previous approaches ^113– 116^.

These approaches have not yet been used to model cortical adaptation, but we believe there is potential. To address adaptation, one would need to consider how learning in deep neural network models is updated over time as the network is exposed to new stimuli. We believe there are two potential routes for achieving this, both of which require extending the deep neural network framework. One approach includes adding recurrent connections that are updated over time. This form of model has interestingly been shown to be effective for modeling contrast adaptation of retinal neurons to natural scenes ^117^. A second approach includes adding divisive normalization to deep neural network models. Divisive normalization is already present in simple forms in deep convolutional neural networks (see e.g. references in 37, 118). One would need to extend such models to incorporate adaptation in time. We should emphasize that the potential of incorporating adaptation into deep neural networks is not limited to supervised discriminative networks. There has also been recent progress in building unsupervised deep convolutional networks with divisive normalization (e.g. 119). Deep convolutional networks together with more plausible non-linearities may provide a means of modeling adaptation in cortical neural areas beyond V1 but will ultimately need to be tested against experimental data.

In summary, simple stimuli were the starting point for studying the visual system and have taught us much over the past 50-plus years, but, as a field, our focus should shift to more complex stimuli. We suggest that experiments and models focus on using natural stimuli for both learning and testing for two reasons: 1) the greater complexity in natural inputs as compared to simple stimuli will increase our understanding of how the visual cortex deals with this complexity, potentially leading to the discovery of new phenomena, and 2) a better understanding of how cortex processes natural images could potentially equip us with more powerful tools for computer vision.

## Summary

Our ability to record and predict neuronal and perceptual responses has improved dramatically over the last few years. We are able to record from more neurons simultaneously, using more complex stimuli, over longer periods of time. As we have elucidated more and more about how adaptation occurs, this has informed our understanding of the properties of adaptation. In parallel developments, theory has helped formulate hypotheses for the functional role of adaptation. In sum, it is our hope that increased capabilities for studying visual cortex at the population level, and with more naturalistic stimuli, combined with the interplay with modeling approaches, will help move forward our understanding of adaptation and its functional goals.
